# Influence of early rehabilitation nursing on postoperative rehabilitation of patients with hallux valgus based on information-motivation-behavior skills model

**DOI:** 10.12669/pjms.41.1.9791

**Published:** 2025-01

**Authors:** Shuting Yu, Yiqin Yang, Hui Su, Ling Zhou, Jin Zhou

**Affiliations:** 1 Shuting Yu, Department of Nursing, The Sixth People’s Hospital Affiliated to Shanghai Jiaotong University, School of Medicine, Shanghai 200000, China; 2 Yiqin Yang, Department of Nursing, The Sixth People’s Hospital Affiliated to Shanghai Jiaotong University, School of Medicine, Shanghai 200000, China; 3 Hui Su, Department of Nursing, The Sixth People’s Hospital Affiliated to Shanghai Jiaotong University, School of Medicine, Shanghai 200000, China; 4 Ling Zhou, Department of Nursing, The Sixth People’s Hospital Affiliated to Shanghai Jiaotong University, School of Medicine, Shanghai 200000, China; 5 Jin Zhou, Department of Nursing, The Sixth People’s Hospital Affiliated to Shanghai Jiaotong University, School of Medicine, Shanghai 200000, China

**Keywords:** Hallux valgus, Early rehabilitation nursing, Information-motivation-behavior skills model, Postoperative rehabilitation

## Abstract

**Objective:**

To probe the influence of early rehabilitation nursing on postoperative rehabilitation of patients with hallux valgus on the basis of information-motivation-behavior skills (IMB) model.

**Methods:**

Convenience sampling was adopted, and 80 patients with hallux valgus admitted to the Sixth People’s Hospital Affiliated to Shanghai Jiaotong University from July 2020 to July 2022 were randomly separated into control group (CG) and observation group (OG) with 40 patients in each group according to the time of admission. Patients in the CG received routine nursing and rehabilitation guidance and follow-up. Patients in the OG were given rehabilitation guidance and follow-up based on IMB model. The American Orthopaedic Foot and Ankle Association Scoring System (AOFAS) scores, Self-efficacy for Rehabilitation Out-come Scale (SER) scores, The Self-rating Anxiety Scale (SAS) and Self-rating Depression Scale (SDS) scores, quality of Life scores, incidence of complications as well as nursing satisfaction in both groups were compared.

**Results:**

After receiving usual nursing and rehabilitation guidance in each group, AOFAS scores, SER scores, quality of life scores and nursing satisfaction in the OG were elevated compared to the CG (P<0.05), SAS and SDS scores, and the incidence of complications in the OG were lessened compared to the CG (P<0.05).

**Conclusion:**

To sum up, Early rehabilitation nursing based on IMB model could promote the postoperative recovery of limb function after hallux valgus, reduce bad emotions, decrease the occurrence of complications, promote self-efficacy, and promote the quality of life of patients, which is valuable for promotion.

## INTRODUCTION

Hallux valgus, also known as hallux toe synovial cyst, is the most common foot deformity in foot and ankle surgery.^1^ The main features of bunion are excessive inclination of the toe to the lateral side of the foot and the deformity of the forefoot with adduction of the first phalangeal bone, which greatly influences the movement and life of patients.^2^,^3^ With the improvement of life quality as well as the development of medical technology, more and more patients are receiving hallux valgus surgery.^4^,^5^ However, most patients with hallux valgus lack of attention to postoperative rehabilitation exercise, and are resistant to postoperative rehabilitation exercises due to the fear of pain, which has a direct negative impact on surgical efficacy.^6^ Some studies have shown that^7^, good early rehabilitation exercise and self-management for patients with hallux valgus after surgery can effectively improve joint function and reduce the occurrence of various complications. Therefore, relying only on traditional nursing methods can no longer meet the rehabilitation needs of patients, and higher-level postoperative rehabilitation and nursing strategies for bunion patients need to be proposed.

Information-motivation-behavioral skills (IMB) model belongs to a behavioral change theory first proposed by Fisher et al. in the study of acquired immune deficiency syndrome (AIDS) behavioral change in 1992. 8 The model assumes that patients must be informed, motivated, and have the behavioral skills to maintain positive health behaviors.^9^ In recent years, this model was applied to intervention in multiple fields of patient behavior change in foreign countries.^10^ The IMB model yields effectiveness in changing the behavior of patients with type 2 diabetes 11 and patients undergoing coronary artery transplantation.^12^ In addition, Zhao Yuan et al.^13^ found that the implementation of the IMB model could not only significantly promote the recovery of joint function and relieve pain, but also improve the professional skills and professional level of nurses, promote the communication between nurses and patients, and continuously improve the satisfaction of patients.

Although there are many studies on the IMB model at home and abroad, due to the late start in China, there is not much research and practice on the nursing model based on this modelaty. At the same time, there are some difficulties in the implementation process, such as patients cannot adhere to rehabilitation exercise, poor compliance, and difficult return visits.^14^ Based on this, in this study, the model of IMB was applied to the early postoperative rehabilitation exercise of patients with hallux valgus for the first time, and the application effect of the model to change the self-management behavior and rehabilitation effect of patients with hallux valgus was discussed from the three aspects of information, motivation and behavioral skill, so as to provide a new basis for the rehabilitation practice after hallux valgus.

## METHODS

Convenience sampling was adopted, and 80 patients with hallux valgus admitted from July 2020 to July 2022 were randomly divided into control group (CG) and observation group (OG) with 40 patients in each group according to the time of admission. No difference was discovered in the general data between both groups (P>0.05, Table-I).

**Table-I:** General data of patients in both groups.

Items		Control group (n=40)	Observation group (n=40)	P
Average age (years)		43.25±4.35	43.32±4.38	>0.05
Gender (male/female)		18/22	19/21	>0.05
Average course of disease (years)		3.22±1.03	3.25±1.12	>0.05
Degree of hallux valgus	Moderate	16	15	>0.05
	Severe	24	25	
Degree of education	Senior high school	5	6
	Junior college	16	17	>0.05
	Undergraduate	19	17	
Family history		15	16	>0.05
Medical insurance situation		25	27	>0.05

### Ethics Approval:

This study was approved by the Medical Ethical Committee of the Sixth People’s Hospital Affiliated to Shanghai Jiaotong University School of Medicine under the approval number of 2023-KY-037 (K).

### Inclusion criteria:

- Accordance with the diagnostic criteria of hallux valgus.
- Age 25-55 years old, gender unlimited.
- In line with the indications of thumb valgus surgery.
- Good understanding ability and clear consciousness.
- Voluntary participation in the study with informed consent and cooperation in the completion of the study.

### Exclusion criteria:

- Patients with severe cardiovascular along with cerebrovascular diseases.
- Severe mental disorders.
- Heart, liver and kidney insufficiency.
- Patients who could not routinely cooperate with nursing and treatment after surgery.

### Interventions:

Patients in the CG adopted routine nursing and rehabilitation guidance and follow-up: from the second day after admission, patients received routine nursing and rehabilitation guidance, which mainly aimed at different stages of joint function exercise knowledge, health education was carried out by the responsible nurse combined with “Rehabilitation training Manual after thumb valve.” One month after discharge, the responsible nurse would conduct telephone follow-up (once every two weeks) to understand the patient’s recovery at home, urge the patient to visit the outpatient clinic regularly for follow-up, and provide relevant explanations and guidance for the problems raised by the patient.

Patients in the OG were given rehabilitation guidance and follow-up based on IMB model: based on the conceptual framework of IMB model, relevant contents were designed around information support, motivational interview and behavioral skills, and combined with the existing rehabilitation guidance and follow-up plans, the postoperative rehabilitation guidance plan for patients with hallux valgus was developed based on IMB model. The medical and nursing experts in orthopedics and rehabilitation departments were consulted to refine the arrangement of various links during hospitalization and after discharge.

The management team consisted of one orthopaedic specialist nurse, one orthopaedic doctor, one rehabilitation therapist, and two responsible nurses. The team, led by specialist nurses, held regular weekly meetings to discuss or summarize the implementation of rehabilitation guidance programs to achieve quality contro

### Information support intervention.

The basic conditions of patients were evaluated on the second day after admission, including pain, joint function, self-care ability, and social support. The management team developed an individualized information support plan based on the evaluation, and required the patient and his family to participate in decision-making and express their wishes. Before and after operation, responsible nurses provided systematic information support for patients, and timely answered and clarified questions raised by patients. Information intervention was performed for 5-6 times, 10 minutes to 15 minutes each time. (a) *Operation information*: Patients were informed about operation knowledge, including preoperative preparation, anesthesia and operation process, and postoperative management. (b) *Rehabilitation exercise*: The importance of rehabilitation exercise was stressed. Patients were briefly informed the about the rehabilitation exercises. (c) *Psychological nursing*: Nurses carried out individualized guidance for patients’ bad mood before and after surgery, gave psychological support to patients and their families, and established patients’ confidence in recovery. (d) *Guidance for return visit:* Nurses informed the patient to return visit regularly to review the recovery of joint function. After discharge, the patient was followed up by telephone to learn about the patient’s rehabilitation exercise at home. Structured telephone follow-up was adopted. Meanwhile, questions raised by the patient were given timely feedback, and the contents of the call were recorded in the patient’s personal file. The telephone was once a week in the first month, once every two weeks in the second month, along with once a follow-up in the third month. Each call was 10 minutes to 15 minutes.

(2) Motivational interviews were organized by specialist nurses, 8-10 people each time, lasting about 30 minutes, and performed during the patient’s hospitalization and one after discharge. The theme of the first interview was “Understanding of Hallux Ectropion Surgery,” and each patient was encouraged to state his or her own method and postoperative expectations, and the specialist nurse would explain and correct the patient’s misperception or unreasonable expectations. The theme of the second interview was “self-rehabilitation management after thumb valgus surgery,” which clarified the importance of self-rehabilitation exercise, strengthened patients’ awareness of self-management, and guided patients to set phased self-management goals. The third interview was arranged in the second week after the patient was discharged from the hospital. The theme was “Life after bunion valgus,” which encouraged the patients to exchange experience in daily life, transmitted positive emotional experience, and established the confidence of the patients to recover soon.

(3) *Behavioral skills training*: Responsible nurses conducted behavioral skills training at the bedside to strengthen patients’ rehabilitation skills through various methods. (a) Functional exercise guidance: Bedside guidance in stages, including muscle strength training, joint training, standing training, walking training, resistance joint training, and self-care ability training. The responsible nurse demonstrated and guided the patient with videos and until the patient had mastered the content of functional exercise in each stage. The responsible nurse shared relevant information with patients and their families through wechat. (b) Nurses formulated rehabilitation exercise plan and issued rehabilitation exercise schedule, which included two parts: hospital and home. The schedule was listed as the date, exercise items, and frequency. The rehabilitation exercise plan during hospitalization was jointly formulated by the nurse in charge, the patient and his family members, and the patient was supervised to complete it every day and recorded on the schedule. The rehabilitation exercise plan after discharge was formulated by the patient and his family, and the family recorded the patient’s daily completion.

### Observation indexes:

(1) American Orthopaedic Foot and Ankle Association Scoring System (AOFAS)^15^: This scale involved nine scoring items such as pain, functional and voluntary activity, support, maximum walking distance, ground walking, abnormal gait, and foot force line. Higher score indicated better joint function. (2) Self efficacy for Rehabilitation Out-come Scale (SER)^16^: This scale includes two dimensions, task self-efficacy, and coping self-efficacy, with 12 items, and the total score was 0-120. A higher score indicated a higher level of self-efficacy. The Chinese version of the questionnaire had good reliability and validity, with Cronbach’s α being 0.942. (3) The Self-rating Anxiety Scale (SAS) and Self-rating Depression Scale (SDS)^17^ were implemented to evaluate the anxiety and depression of patients. (4) The complications such as thumb stiffness, foot infection, and toe deflection were recorded in both groups during nursing. (5) The Quality of Life questionnaire-core 30 (QLQ-C30)^18^ was used for assessment. The scale included five dimensions: role, social, cognitive, emotional, and physical. Higher score represented better quality of life. (6) Nursing satisfaction was evaluated by the nursing satisfaction scale made by our hospital. The score range was 0-100 points, with more than 80 points being very satisfied, 60-80 points being generally satisfied, and less than 60 points being dissatisfied. Satisfaction = (very satisfied cases + generally satisfied cases)/total cases ×100.00%.

### Statistical analysis:

Data were analyzed by SPSS20.0 software, and measurement data were described by mean ± standard (x±s). Counting data are described by frequency and percentage. For baseline data, chi-square test was used for counting data and independent T-test was used for measurement data. For intervention effect, repeated measure analysis of variance was used. P<0.05 was significant.

## RESULTS

**Fig.1:**
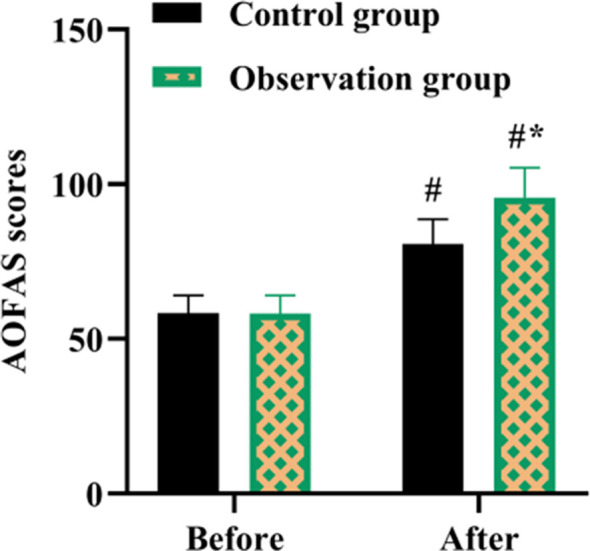
AOFAS scores in both groups. #P<0.05, compared with before nursing. *P<0.05, compared with the control group.

A total of 80 patients who met the inclusion criteria were analyzed in this study. Among them, 40 patients (CG) were treated with routine nursing and rehabilitation guidance and follow-up, and 40 patients (OG) were treated with rehabilitation guidance and follow-up based on the IMB model. Previous to nursing, no difference was discovered in AOFAS scores in both groups (P>0.05). After nursing, AOFAS scores were elevated in both groups, and those in the OG were higher compared to the CG (P<0.05, Fig.1).

**Fig.2:**
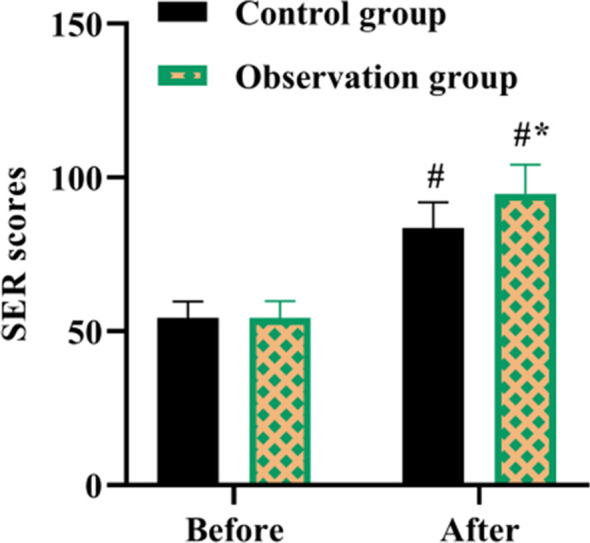
SER scores in both groups. #P<0.05, compared with before nursing, *P<0.05, compared with the control group.

There was no statistically significant difference in SER scores between the two groups before receiving nursing intervention (P>0.05). After treatment, SER scores were elevated in both groups, however the SER scores of OG was significantly higher than that of CG (P<0.05, Fig.2).

**Fig.3:**
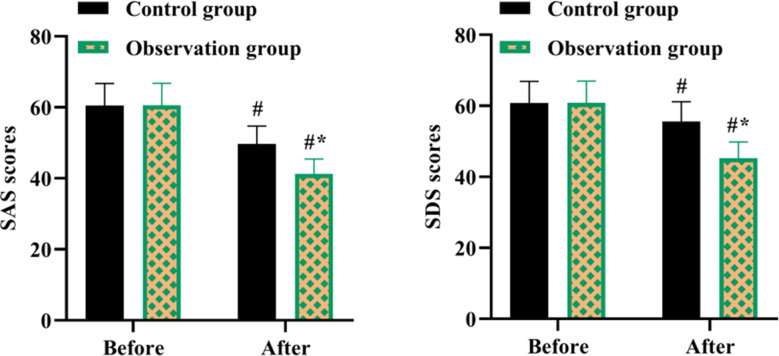
SAS and SDS scores in both groups. #P<0.05, compared with before nursing, *P<0.05, compared with the control group.

There was no statistically significant difference between the two groups in SAS and SDS scores before the nursing intervention (P>0.05). After nursing, both SAS and SDS scores decreased significantly, and the OG score was significantly lower than CG (P<0.05, Fig.3).

**Fig.4:**
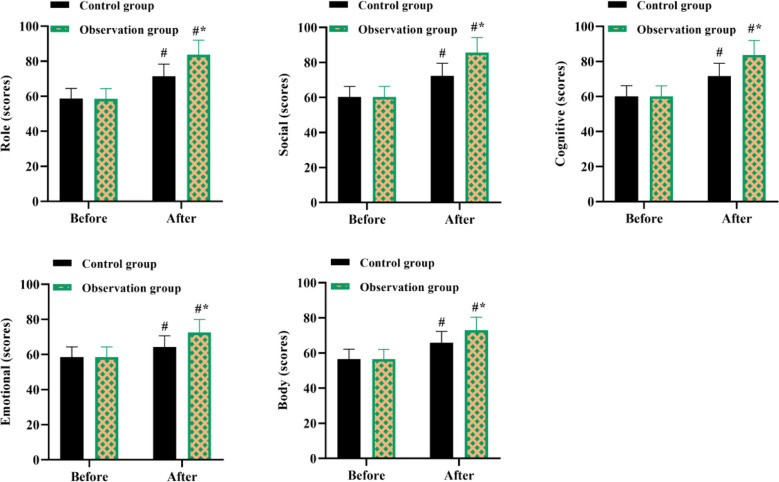
five dimensions (role, social, cognitive, emotional, and physical) of Quality of life scores in both groups. #P<0.05, compared with before nursing, *P<0.05, compared with the control group.

**Fig.5:**
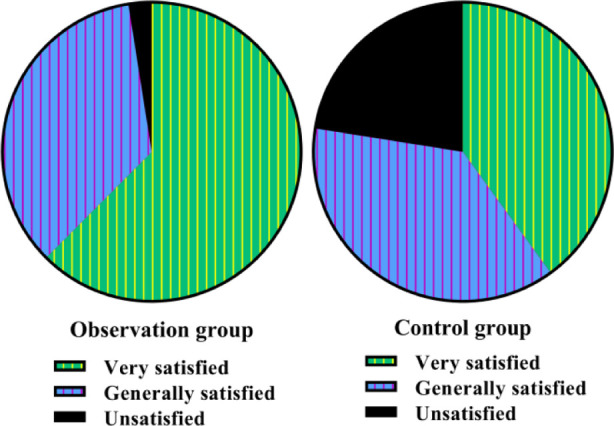
Nursing satisfaction in both groups. Nursing satisfaction: Very satisfied, General satisfaction and Unsatisfied.

We also counted postoperative complications of hallux valgus, including thumb stiffness, foot infection, and toe deflection. The results showed a significantly lower complication rate with OG compared with CG (P<0.05, Table-II). Before nursing, no difference was discovered five dimensions (role, social, cognitive, emotional, and physical) of quality-of-life scores in both groups (P>0.05). After nursing, five dimensions of quality of life scores were elevated in both groups, and those in the OG were higher compared to the CG (P<0.05, Fig.4). In addition, the nursing satisfaction of patients in the OG was significantly higher than in the CG (χ^2^=7.31, P<0.05, Fig.5).

**Table-II:** Incidence of complications in both groups (n, %).

Groups	Thumb stiffness	Foot infection	Toe deflection	Incidence rate
Control group (n=40)	2	3	2	7 (17.50%)
Observation group (n=40)	0	1	0	1 (2.50%)
*χ* ^2^	5.00			
*P*	<0.05			

## DISCUSSION

The results of this study suggest that rehabilitation interventions based on the IMB model can lead to better, faster, and more effective recovery compared with conventional care and rehabilitation interventions.^19^ Hallux valgus is caused by congenital and acquired poor posture, and the normal life and work of severe patients can be greatly influenced.^20^ Currently, the treatment of hallux valgus is mainly based on surgery, and postoperative nursing and rehabilitation guidance for the prognosis of hallux valgus has a very important role.^21^ Several previous studies have assessed postoperative clinical outcomes by means of the AOFAS score.^6^,^22^ In this study, the AOFAS score of OG was significantly higher and the incidence of complications was lower, which suggests that the intervention of the IMB model can better improve the postoperative limb function after bunion surgery, as well as assist the effect of physical therapy.

Besides, the recovery of postoperative joint function is not only linked to the operative effect, but also linked to the patient’s compliance with postoperative rehabilitation nursing, which helps patients to mobilize early postoperative rehabilitative therapy and functional exercise.^23^ Elif Bakır et al. pointed out that IMB model-based intervention promoted glycemic control among adolescents with type 1 diabetes.^24^ A study proposed by Wei Shen et al. discovered that IMB-based continuous nursing care effectively promoted medication compliance, enhanced self-care ability, as well as promoted the quality of life of patients with aplastic anemia.^25^ Meihua Li et al. found that an IMB model intervention in patients with coronary artery disease undergoing cardiac rehabilitation facilitated improvement in cardiorespiratory fitness and enhanced self-efficacy.^26^ Our results are consistent with these observations. We tested patients’ self-efficacy for postoperative rehabilitation by means of the SER scores. Improved self-efficacy can increase adherence to postoperative rehabilitation exercises and promote patient recovery. SER scores, SAS and SDS scores, quality of life scores, and satisfaction with care were elevated in OG compared with CG.

Different from the traditional nursing mode, rehabilitation nursing on the basis of the IMB model is more conducive to the communication between nurses and patients. Patients can develop a positive belief in rehabilitation, gain a thorough understanding of postoperative disease and rehabilitation exercises, and significantly increase their enthusiasm and initiative to engage in rehabilitation nursing activities, which will boost their confidence in rehabilitation exercises.^26^ In addition, motivational interviews after discharge are conducted to encourage patients to communicate their feelings and ideas, help them establish reasonable expectations of postoperative effects, and feel the benefits brought by positive postoperative rehabilitation behaviors, so as to promote the formation and maintenance of behavioral changes.^27^ Therefore, early rehabilitation nursing based on the IMB model could promote the postoperative recovery of limb function after hallux valgus, reduce bad emotions, decrease the incidence of complications, promote self-efficacy, and promote the quality of life of patients. This model analyzed problems from different angles, saved time and labor, but also saved medical costs, could make up for the shortcomings of the traditional working model, was conducive to the formulation and implementation of early postoperative rehabilitation nursing program for patients, and could expand the occupation category of each sub-professional staff. It had extensive social and economic benefits and was worth popularizing.

### Limitation of the study:

The sample size of this study was from a single center and the number of patients included was limited. Therefore, bias may be present. We will continue to expand the clinical sample size in the future to provide more rigorous data. In addition, this study did not analyze the long-term rehabilitation of patients after hallux valgus, which still needs to be explored.

## CONCLUSION

Our studies have shown that early rehabilitation nursing based on the IMB model can enhance the recovery of limb function after hallux valgus, prevent complications, improve self- efficacy and improve patients’ quality of life. It also provides a new approach and clinical rationale for the postoperative clinical rehabilitation and nursing of hallux valgus, as well as provides enlightenment for the IMB model in the rehabilitation of other related diseases.

### Author’s Contribution:

**SY and JZ:** Conceived and designed the study, literature search.

**YY, HS and LZ:** Collected and managed the data, analyzed the data of the study.

**SY:** Literature search, Prepared the manuscript.

**JZ:** Participated in the final revision,

All authors have read the final version and are responsible and accountable for the accuracy or integrity of the work.
